# Intergenerational Transmission of Family Violence: A Narrative Review of Pathways from Childhood Exposure to Family Violence to Adult Intimate Partner Violence Perpetration

**DOI:** 10.3390/bs16020299

**Published:** 2026-02-20

**Authors:** Sejung Yang, Yangjin Park, Pa Thor

**Affiliations:** 1 Department of Social Work, Ohio University, Athens, OH 45701, USA; 2 School of Social Work, University of Texas at Arlington, Arlington, TX 76019, USA; yangjin.park@uta.edu; 3 Department of Anesthesiology, Hospital for Special Surgery, New York, NY 10021, USA; thorp@hss.edu

**Keywords:** child abuse, childhood exposure to family violence, exposure to intimate partner violence, intergenerational transmission of family violence, intimate partner violence perpetration

## Abstract

Purpose: A substantial body of research indicates that exposure to violence during childhood is linked to long-term harmful effects. More specifically, child abuse and exposure to parental intimate partner violence (IPV) may increase the likelihood of IPV perpetration in adulthood. This narrative review integrates theoretical and empirical perspectives to provide a comprehensive understanding of the intergenerational transmission of family violence, while identifying gaps in the literature and suggesting directions for future research. Methods: Relevant peer-reviewed empirical studies were identified through major academic databases and reference searches, with a focus on research addressing pathways from childhood exposure to family violence (CEFV) to adult IPV perpetration. The review synthesizes empirical findings to consolidate current knowledge and identify areas for further investigation. Findings: Existing studies have extensively examined associations between CEFV and adult IPV perpetration based on various theoretical frameworks, such as social learning theory, emotional regulation perspective, and the adverse childhood experiences framework. Collectively, these theoretical perspectives underscore that intergenerational transmission of family violence is shaped by behavioral, emotional, cognitive, and contextual factors. However, most studies have focused predominantly on individual and familial characteristics, with limited attention to community or broader socioecological influences. Furthermore, most of the studies have primarily been grounded in the victim–perpetrator binary framework, which treats IPV perpetration and victimization as distinct phenomena. Multidimensional aspects of violence and abuse, such as duration, severity, context, and frequency, also remain underexplored. Conclusions: This review underscores the need to (1) examine the roles of socioecological factors in the intergenerational transmission of family violence, (2) shift the paradigm beyond the gendered victim–offender binary, (3) account for the multifaceted nature of violence and abuse, and (4) utilize diverse methodological approaches to advance the field.

## 1. Introduction

Intimate partner violence (IPV) is a pervasive social problem and a significant public health concern. In the United States, 47.3% of women and 44.2% of men have reported experiencing sexual violence, physical violence, or stalking by an intimate partner in their lifetime (Leemis et al., 2022). The negative impacts of IPV are far-reaching, influencing victims’ physical and mental health (Dekel et al., 2019; Hullenaar et al., 2022; Ruork et al., 2022; Wadsworth et al., 2018), education (Adams et al., 2013), employment (Swanberg et al., 2006), and financial stability (Blodgett & Lanigan, 2018; Peterson et al., 2018). IPV significantly deteriorates a victim’s physical and mental health (Dekel et al., 2019; Hullenaar et al., 2022; Ruork et al., 2022; Wadsworth et al., 2018). For example, IPV victimization has been linked to conditions such as post-traumatic stress disorder and depression (Dekel et al., 2019; Ruork et al., 2022). Furthermore, research indicates that women who have experienced IPV often achieve lower levels of education than their counterparts, which subsequently leads to reduced income (Adams et al., 2013). IPV victims may struggle to maintain long-term employment, frequently being forced to leave jobs for their safety (Swanberg et al., 2006). The financial burden of IPV is also substantial. Peterson et al. (2018) estimated the average lifetime cost of IPV to exceed $100,000 for women and $20,000 for men, encompassing medical expenses, lost productivity, and property damage.

IPV has a profound impact beyond the individuals directly involved, significantly impacting children exposed to it. The detrimental effects of IPV on children include increased internalizing and externalizing behaviors, impaired socio-emotional functioning, deteriorated well-being, and reduced life satisfaction in adulthood (Riina, 2021; Arslan, 2016; Greger et al., 2017; LaBrenz et al., 2021). For example, exposure during infancy has been linked to poorer social skills and higher peer victimization by age ten (Schulz et al., 2024). The social–emotional competence of children exposed to IPV is frequently compromised, with systematic reviews reporting higher rates of peer problems, bullying, and relational difficulties (Bender et al., 2022). The long-term consequences are profound as childhood exposure to parental IPV is associated with double the risk for perpetrating emotional IPV in adulthood (Osborne et al., 2025).

Moreover, when caregivers are entangled in interpersonal conflicts, children’s vital developmental needs, such as secure attachment (McIntosh et al., 2021) and consistent family routines (Romano et al., 2022), are often neglected. Research further indicates that the relationship between IPV exposure and children’s internalizing and externalizing problems is mediated by parenting and parental mental health, with child temperament moderating outcomes (Lee et al., 2024). Exposure to IPV can also undermine children’s effortful control, referring to the capacity to regulate attention, inhibit impulsive responses, and adjust behavior and emotions following situational demands (Clark & Hankin, 2024). Exposure to IPV can compromise children’s effortful control by disrupting key developmental environments of children, especially caregiver functioning. IPV affects not only children directly but also the family environment, including caregivers’ psychological well-being (Romano et al., 2022). In particular, exposure to IPV appears to alter the link between caregiver depressive symptoms and children’s effortful control, such that higher caregiver depression is more strongly linked to lower effortful control among IPV-exposed adolescents (Clark & Hankin, 2024). Ultimately, IPV is likely to impact all involved, whether directly or indirectly, including perpetrators, victims, and family members, notably children.

The intergenerational transmission of family violence (ITFV) is a significant factor in explaining adult IPV perpetration (Meinck et al., 2023). Early violent experiences, such as child maltreatment, exposure to parental IPV, or sibling violence, are consistently linked to adverse outcomes later in life. While the term family violence broadly encompasses various forms of intrafamilial violence, such as IPV, child abuse, sibling violence, and elder abuse (Warren et al., 2024), the present review focuses on child exposure to family violence (CEFV) in the form of parental IPV and child abuse as primary antecedents of adult IPV perpetration. To date, numerous studies have established that CEFV has often been linked to children’s future IPV perpetration (Chen et al., 2025; Eriksson & Mazerolle, 2015; Herrenkohl & Jung, 2016; Jung et al., 2019; Lünnemann et al., 2019; Roberts et al., 2010; Roberts et al., 2011; Whitfield et al., 2003). For instance, a 30-year follow-up of 2401 participants from a Queensland, Australia birth cohort found that child abuse and neglect were linked to adult IPV (Jiang et al., 2025). Recent meta-analytic evidence highlights that individuals exposed to emotional IPV in childhood are approximately twice as likely to perpetrate emotional IPV in adulthood, with the risk from emotional violence (OR ≈ 3.03) being higher than that from physical IPV exposure (Osborne et al., 2025). Furthermore, a recent review emphasizes that parental histories of interpersonal violent trauma and associated psychopathology (e.g., maternal PTSD or depression) can have a negative impact on children’s development and the subsequent transmission of violence across generations (Graf & Schechter, 2024).

Despite these insights, it is also marked by notable methodological limitations and critiques, including a heavy reliance on retrospective self-reports, inconsistent definitions of exposure and outcome, and limited longitudinal research (Haselschwerdt et al., 2018). A 2023 scoping review reported that children whose parents experienced adverse childhood experiences (ACEs), including family violence, face an elevated risk of both exposure to violence and later perpetration or victimization (Meinck et al., 2023).

This narrative review specifically focuses on child exposure to family violence (CEFV), particularly parental IPV and child abuse, as key precursors to adult IPV perpetration. By integrating both quantitative and qualitative evidence, we aim to assess methodological rigor, identify key gaps, and provide a refined foundation for future empirical research and the development of evidence-based interventions.

## 2. Methods

This narrative review employed a structured yet interpretive approach to synthesize empirical literature on the ITFV (Greenhalgh et al., 2018; Sukhera, 2022). The primary aim of this review is to integrate multidisciplinary empirical findings to advance conceptual and applied understanding of adult IPV perpetration following CEFV. A narrative approach is particularly appropriate for this study because it allows for a comprehensive exploration of both quantitative and qualitative literature. While systematic reviews are well-suited for synthesizing quantitative evidence, they often exclude qualitative studies and theoretical perspectives that provide nuanced insights into complex phenomena (Greenhalgh et al., 2018). By facilitating interpretation and critical appraisal, a narrative review can synthesize existing research, identify knowledge gaps, and inform future research directions.

To identify relevant studies, a structured literature search was conducted using key terms such as “domestic violence,” “domestic aggression,” “domestic abuse,” “IPV,” “partner violence,” “partner aggression,” “partner abuse,” “dating violence,” “marital violence,” “marital aggression,” “marital abuse,” “marital conflict,” “intergenerational transmission of violence,” “ACEs,” “childhood exposure to family violence,” “child maltreatment,” “child abuse,” “parental IPV,” “parental domestic violence,” “witnessing domestic violence,” and “witnessing IPV”, across major academic databases, including PsycINFO, PubMed, Scopus, and Google Scholar. To ensure comprehensive coverage beyond database searches, the reference lists of relevant articles were manually screened to identify additional quantitative and qualitative studies.

Studies were included if they (a) were peer-reviewed empirical articles, (b) examined CEFV as a predictor, and (c) identified adult IPV perpetration as the outcome. Studies were excluded if they focused exclusively on IPV victimization, teen dating violence, child abuse perpetration, or non-family violence outcomes. Theoretical papers were reviewed selectively to support interpretation but were not treated as primary empirical evidence.

Using these inclusion and exclusion criteria, the studies were screened and reviewed in a structured, yet interpretive manner. First, titles and abstracts were screened for relevance, followed by full-text review to confirm eligibility. A total of 18 studies met the criteria and were included in the review. The selected studies were synthesized using an interpretive thematic narrative approach, with attention to recurring conceptual models, methodological approaches, and theoretical perspectives across studies. This approach enabled the integration of findings across diverse research designs, the identification of knowledge gaps, and the recommendations for future research.

## 3. Findings

A body of quantitative research has examined the association between CEFV and IPV perpetration in adulthood using various theoretical frameworks and analytic approaches. Notably, numerous studies have investigated the potential mediating and moderating mechanisms of the cycle of violence (Meinck et al., 2023). Additionally, patterns of ITFV considering gender or types of CEFV, such as child abuse or parental IPV, have also been examined. In contrast, there are a few qualitative studies that have examined these processes.

### 3.1. Pathways from CEFV to IPV Perpetration

Numerous empirical studies across disciplines have examined mediators and moderators explaining the pathways from CEFV to IPV perpetration (see Table 1). Across studies, several mediating factors were examined between the link between CEFV and IPV, such as social learning processes (Eriksson & Mazerolle, 2015; Markowitz, 2001; Wareham et al., 2009), social information processing (Fite et al., 2008), emotional regulation (Kim et al., 2009; Oliveros & Coleman, 2021), stress sensitization (Roberts et al., 2011), and adolescent externalizing behaviors (Ehrensaft et al., 2003; Low et al., 2019). Other studies have examined CEFV and later IPV perpetration within the ACEs framework, not necessarily investigating specific mediating or moderating mechanisms (Roberts et al., 2011; Voith et al., 2017; Whitfield et al., 2003).

#### 3.1.1. Social Learning Theory

Social learning theory posits that children exposed to family violence tend to learn about violence while observing and imitating the violent behaviors of their parents or caregivers (Bandura, 1973). Parental or caregiver violence provides an influential model for children regarding the usage of violence (Bandura, 1973). Consequently, when children reach adulthood, they are likely to replicate their parents’ or caregivers’ violent behaviors in their intimate relationships (Bell & Naugle, 2008). Several studies have investigated the pathways from CEFV to adulthood IPV based on social learning theory by focusing on potential mediators such as attitudes justifying IPV (Eriksson & Mazerolle, 2015; Markowitz, 2001; Wareham et al., 2009). Based on mixed results from those studies, it is unclear whether holding such attitudes mediates the relationship between CEFV and later IPV perpetration, while the attitude is associated with IPV perpetration. Attitudes toward the use of violence mediated the association between family violence exposure and IPV perpetration among a sample of ex-offenders (*n* = 141) and the general population (*n* = 245; Markowitz, 2001). Wareham et al. (2009) found that the impacts of physical abuse on minor IPV were fully mediated by social learning measures, but severe IPV was not mediated among 204 male family violence perpetrators. Eriksson and Mazerolle (2015) found that while holding attitudes justifying IPV was found to be associated with IPV perpetration, holding such attitudes was not found to mediate the relationship between exposure to parental IPV during childhood and later IPV perpetration among male arrestees (*n* = 303).

These inconsistent findings may be partially due to the small sample size of studies, most of which included male adults involved in the criminal legal system. In sum, although social learning theory offers a valuable framework for understanding the ITFV, the inconsistent empirical findings highlight that this theory alone may not fully explain the pathways from childhood exposure to adult IPV perpetration. Future research should explore additional social, emotional, and contextual factors that interact with learned attitudes to better capture the dynamics of ITFV.

#### 3.1.2. Social Information Processing

Social information processing theory explains the ways in which children with aggressive behaviors identify, evaluate, and make decisions about information in social situations, which increases their likelihood of engaging in behavior problems (Dodge & Crick, 1990). Fite et al. (2008) found partial support for social information processing in ITFV. This study examined four social information processing stages to determine their effects as mediators by using structural equation modeling (SEM). The four social information processing stages include: (1) encoding, or registering social cues; (2) hostile attribution, or interpreting ambiguous cues as hostile or threatening; (3) generation of aggressive responses; and (4) evaluation of aggressive responses, or appraising aggressive reactions as acceptable or effective. A significant mediating effect of the response generation and response evaluation was reported, whereas there was no evidence found on the mediating effect of encoding and attributions. The findings suggest that children’s capability to create diverse social responses and appropriately assess the possible consequences of their responses mediates the link between CEFV and later IPV.

Difficulties in how individuals process and respond to social information, especially in developing and assessing nonviolent responses, appear to contribute to the continuation of violence across generations. However, since not all aspects of social information processing were found to mediate this link, future research should further examine how these cognitive processes interact with emotional and contextual factors in the ITFV.

#### 3.1.3. Emotional Regulation Perspective

Emotional regulation is defined as the processes by which individuals assess and manage their emotional responses (Thompson & Meyer, 2007). Emotion dysregulation was found to be a crucial mediating factor in ITFV (Kim et al., 2009; Oliveros & Coleman, 2021). CEFV may be related to emotional dysregulation in numerous ways. Traumatic experiences can lead to detrimental neurobiological consequences among children (Cicchetti & Rogosch, 2001; Kaufman et al., 2000; D. J. Siegel, 2001). Additionally, those experiencing IPV are likely to have a challenging time taking parental responsibilities and unlikely to meet various child emotional and interpersonal needs, which, in turn, may impact children’s emotional regulation development (J. P. Siegel, 2013).

The impaired emotional regulatory process, which results from family violence exposure, is likely to have unfavorable consequences among children. For instance, emotional dysregulation was found to mediate the association between exposure to parental IPV and the development of internalizing and externalizing behaviors among children (Harding et al., 2013). Kim et al. (2009) used prospective longitudinal data, including males and their parents, to test the role of emotional dysregulation in ITFV. Emotional dysregulation of parents was associated with difficulty in the emotional regulation of sons, which was, in turn, linked with conflicts in the intimate relationships of the sons. Similarly, in a more recent study, exposure to parental IPV was associated with emotional regulation difficulties and current IPV in young adults (475 females, 145 males). In the same study, fathers’ aggressive behaviors were strongly associated with emotional regulation difficulties and aggression in the intimate relationships among adult children (Oliveros & Coleman, 2021).

Overall, these findings suggest that emotion dysregulation serves as an important mechanism linking childhood exposure to family violence and later IPV perpetration, although additional factors such as neurobiological, cognitive, relational, and contextual factors likely interact with this process to shape violent behaviors across generations.

#### 3.1.4. Stress Sensitization Theory

Stress sensitization theory posits that CEFV, one of the crucial adverse childhood experiences (ACEs), can sensitize individuals to react more intensely to stressful events. The theory was tested as a potential mechanism of the relationship between CEFV and IPV perpetration (Roberts et al., 2011). For males with high-level ACEs, past-year stressors were associated with an 8.8% increased likelihood of physical and sexual IPV perpetration compared to a 2.3% increased likelihood among their counterparts with a lower level of ACEs. Females with high-level ACEs had a 14.3% increased risk compared with a 2.5% increased risk among their counterparts. The findings indicate that increased vulnerability and hyperreactivity to stressors may be key factors linked to CEFV with IPV perpetration.

#### 3.1.5. Developmental Psychopathology Framework

Developmental psychopathology focuses on how biological, psychological, and social factors interact across a person’s life to influence mental health and behavior (Cicchetti & Rogosch, 2002). Two longitudinal prospective studies suggest that adolescent psychopathological symptoms, particularly externalizing behavior problems, may mediate the relationship between CEFV and later IPV perpetration. One study, conducted by Ehrensaft et al. (2003), followed 543 children over 20 years and found that adolescent behavioral problems appeared to mediate the relationship between CEFV and IPV perpetration in adulthood. Similarly, the more recent longitudinal study (*n* = 205) also found that adolescent externalizing behavior problems, out of three indices of the psychopathology symptoms (i.e., internalizing, externalizing, and combined), mediated the link between childhood exposure to parental IPV and young adult IPV perpetration (Low et al., 2019).

Overall, these findings indicate that adolescent psychopathology, particularly externalizing behavior problems, serves as an important developmental pathway linking CEFV with later IPV perpetration, highlighting the role of early emotional and behavioral difficulties in the ITFV.

#### 3.1.6. Adverse Childhood Experiences Framework

ACEs research has focused on an enduring, harmful impact of a wide range of detrimental childhood experiences on individuals’ health throughout their lives (Voith et al., 2017). To date, ACEs research has extensively investigated the longitudinal associations between ACEs and later various behavioral health outcomes (Roberts et al., 2011; Roberts et al., 2011; Simkin et al., 2025; Voith et al., 2017; Whitfield et al., 2003). Several studies examined the relationship between CEFV and later IPV within the framework of ACEs, as CEFV is a major adverse and traumatic experience, and IPV is one form of critical behavioral health outcomes (Roberts et al., 2011; Voith et al., 2017; Whitfield et al., 2003). For example, physical abuse, emotional abuse, and sexual abuse were associated with IPV perpetration for both males and females in multivariate models including all other childhood adversities (*n* = 34,653). Additionally, among individuals who had all three forms of violent childhood experiences, the risk of victimization was increased 3.5-fold for women, and that of perpetration was increased 3.8-fold for men (*n* = 8629, Whitfield et al., 2003). Similarly, college men reporting child physical abuse were at increased risk of perpetrating psychological IPV (*n* = 423; Voith et al., 2017).

### 3.2. Patterns of ITFV

Numerous studies have explored whether ITFV occurs in a gender-specific, role-specific, or generalized manner (see Table 2; Avakame, 1998; Chen et al., 2025; Eriksson & Mazerolle, 2015; Jung et al., 2019; Kalmuss, 1984; Kwong et al., 2003). Gender-specific modeling posits that children model their same-sex parents’ role, indicating male-to-female violence would be associated with males’ IPV perpetration and females’ IPV victimization (Kwong et al., 2003). Role-specific modeling explains that children’s exposure to IPV has greater predictive power than child abuse in terms of IPV perpetration (Kalmuss, 1984). Kalmuss (1984) reported that, compared to experiencing child abuse, exposure to parental IPV could be more detrimental to IPV engagement in adulthood. In contrast, generalized modeling indicates that family violence in general conveys to children that violence among family members is acceptable, which in turn increases the likelihood of children experiencing any type of family violence in later life (Avakame, 1998; Kwong et al., 2003). For instance, both Avakame (1998) and Kwong et al. (2003) conducted studies that found no indication of specific patterns such that all forms of CEFV were associated with all forms of adulthood IPV.

More recently, Jung et al. (2019) examined gender differences in the association between CEFV and adult IPV perpetration and victimization. Childhood exposure to physical and emotional abuse in boys was likely to be associated with multiple types of IPV, including intimidation. At the same time, girls were associated with physical violence with lower intimidation. One limitation of this study is that the analysis did not distinguish between IPV perpetration and victimization. Similarly, Chen et al. (2025) reported that childhood physical abuse directly predicted adult IPV perpetration among males, while childhood physical abuse was associated with IPV psychological victimization indirectly through psychological distress among females. Unlike the gender-specific modeling framework, which assumes that children imitate their same-sex parents’ roles, these studies focused on examining whether the effects of CEFV differ by gender.

Mixed findings on ITFV patterns may be due to the inconsistent and varying measurements and multifaceted aspects of violence. Furthermore, various dimensions of violence, such as severity, duration, frequency, and context of violence, should be considered in empirical studies, which may be critical and lead to adulthood IPV. For example, child abuse by a father may be more severe than child abuse by a mother, or the period of exposure to parental IPV may be longer than that of child abuse. Regardless, these findings collectively support the ITFV.

### 3.3. Qualitative Insights

There has been limited research on ITFV, specifically pathways from CEFV to IPV perpetration, using the qualitative approach in this field. Antle et al. (2020) explored childhood exposure to parental IPV and participants’ beliefs, attitudes, and engagement in their own IPV experiences. Although participants agreed that violence in intimate and family relationships should not be tolerable, several adolescents admitted that their exposure to parental IPV had influenced them to make them more tolerant of abusive behaviors in their intimate relationships. This finding seems to support the proposition of the widely regarded social learning theory, which posits that children exposed to parental IPV are likely to believe that using violence within the family is justified and legitimized.

Another qualitative study explored the ITFV in Peruvian families through semi-structured interviews with adolescents, mothers, and grandparents from nine families. The study identified three main themes: (1) factors that perpetuate violence, including rigid gender roles, exposure to violence, irritability, violent parenting, alcohol use, and economic dependence; (2) protective factors, such as support networks, overcoming trauma, education, knowing how to choose a partner, and separation from the abuser; and (3) religious beliefs, which can both support coping and perpetuate tolerance of violence. The findings highlight the interplay of individual, relational, community, and societal levels, consistent with the socio-ecological model. Interventions should address both victims and aggressors, promote positive role models, strengthen social support, and challenge cultural norms like machismo and marianismo. Limitations include a predominance of female participants, challenges in recruitment, and similar educational backgrounds, which may affect generalizability (Vilches et al., 2025).

While qualitative research on ITFV is still limited, the available evidence consistently provides support for the ITFV. Antle et al. (2020) found that adolescents exposed to parental IPV often developed greater tolerance for abusive behaviors. Similarly, Vilches et al. (2025) identified factors that perpetuate or mitigate violence in Peruvian families. These findings underscore the need for further qualitative studies to explore how beliefs about CEFV influence later IPV experiences.

## 4. Conceptual and Methodological Gaps

To date, the associations between CEFV and adulthood IPV, considering gender, types of family violence experiences (i.e., exposure to parental IPV and child abuse), and dose–response relationships, have been extensively examined (Avakame, 1998; Chen et al., 2025; Eriksson & Mazerolle, 2015; Jung et al., 2019; Kalmuss, 1984; Kwong et al., 2003). Numerous studies have also investigated mediating and moderating mechanisms of the cycle of violence (Avakame, 1998; Ehrensaft et al., 2003; Eriksson & Mazerolle, 2015; Fite et al., 2008; Whitfield et al., 2003; Kim et al., 2009; Low et al., 2019; Markowitz, 2001; Oliveros & Coleman, 2021; Roberts et al., 2011; Wareham et al., 2009). Limited research, however, has explored perspectives on ITFV using a qualitative approach (Antle et al., 2020; Vilches et al., 2025). Based on the investigation, this review identified several conceptual and methodological gaps in the literature.

### 4.1. Victim–Offender Binary and Gendered Norms

Overall, studies have primarily focused on males who perpetrated IPV unidirectionally. Exposure to parental IPV has also been conceptualized as exposure to male-to-female IPV in childhood and has been mostly assessed as exposure to male-to-female IPV (i.e., father or mother’s partner perpetrating IPV to mother; Roberts et al., 2010; Roberts et al., 2011; Whitfield et al., 2003). Additionally, several studies on ITFV have examined the relationship between CEFV and IPV perpetration for males and victimization for females, presuming males as perpetrators and females as victims (Roberts et al., 2010; Whitfield et al., 2003). This trend may be explained by the fact that, in general, males are likely to be involved in severe physical IPV (Taft et al., 2009), and females experience more fear and injuries from IPV victimization (Black et al., 2010; Gerber et al., 2014). Recent research has begun to include instances of female-perpetrated violence (Chen et al., 2025; Jung et al., 2019).

### 4.2. Multidimensional Aspect of Violence

Most studies on ITFV have focused on physical violence with a few recent exceptions (Chen et al., 2025; Jung et al., 2019). Future studies should include distinct types of violence to fully understand the whole picture of ITFV. For instance, to fill the gap in the current research, studies need to address diverse types of violence, such as childhood sexual abuse, psychological abuse, neglect, or exposure to parental IPV. This research should seek to explore the associations between these types of violence and various kinds of adulthood IPV experiences. While a few studies have assessed the frequency or severity of violence (Wareham et al., 2009; Whitfield et al., 2003), there is still little consideration of other aspects of violence, such as the onset of the incident and chronicity of CEFV. For instance, dichotomous questions were often used to measure child abuse and exposure to IPV (Eriksson & Mazerolle, 2015; Franklin & Kercher, 2012; Kwong et al., 2003; Roberts et al., 2010), which may oversimplify the complexity of individuals’ experiences and obscure differences in other dimensions of violence, such as severity, duration, and timing.

### 4.3. Co-Occurrence of Multiple Forms of Family Violence

Numerous studies have found evidence of the co-occurrence of multiple types of child abuse, including child abuse and exposure to parental IPV (Gutowski & Goodman, 2020; Kelleher et al., 2006; Knickerbocker et al., 2007; Slep & O’Leary, 2005). The rate of adolescents who are often both exposed to IPV and abused by their parents is about 80% (Saunders, 2003). Nevertheless, numerous research studies on ITFV have not systematically addressed their co-occurrence. Future research needs to address the co-occurrence of family violence to fill this gap in the current ITFV literature.

### 4.4. Methodological Considerations

Numerous prospective studies since the early 2000s have been conducted to examine the link between experiencing family violence during childhood and future IPV involvement (Ehrensaft et al., 2003; Fite et al., 2008; Jung et al., 2019; Kim et al., 2009). More longitudinal study designs should continue to be employed to capture the multilayered aspects of violence during childhood (e.g., developmental timing, duration, and severity) in the analysis to enhance the methodological validity of the research.

Concerning types of samples, some studies have used a representative sample (Kalmuss, 1984; Markowitz, 2001; Roberts et al., 2011), whereas others have used selective samples such as male arrestees (Eriksson & Mazerolle, 2015), male domestic violence offenders (Wareham et al., 2009) or college students (Carr & VanDeusen, 2002; Voith et al., 2017). When using a selective sample, it is important to exercise caution when interpreting the results and suggesting implications for intervention. Also, the majority of study participants in the literature have been predominantly heterosexual (Carr & VanDeusen, 2002; Jung et al., 2019; Voith et al., 2017). Future research should include more diverse samples in terms of sexual orientation, gender identity, and other demographic characteristics to enhance the generalizability of the findings (Black et al., 2010; Ehrensaft et al., 2003).

## 5. Recommendations for Future Studies

Prior studies on ITFV have primarily examined male-to-female IPV and physical forms of violence; a comprehensive understanding of ITFV requires attention to assumptions about victims and offenders regarding gender norms, diverse types and dimensions of violence, and the co-occurrence of multiple forms of family violence, as well as methodological considerations, including sample selection and study design. This review suggests several recommendations for future research.

### 5.1. Paradigm Shift from Gendered Victim–Offender Binary Framework

Regarding the conceptualization of IPV, most studies on ITFV seem to treat perpetration and victimization as independent phenomena and examine them separately (Jung et al., 2019; Voith et al., 2017). Studies have primarily been based on the victim–perpetrator binary premise and have considered IPV perpetration and victimization as separate matters. Consequently, most studies have overlooked the potential overlap between IPV perpetration and victimization, where one person can both cause harm and be harmed. Given a growing body of research indicating the overlap or bi-directional aspect of IPV perpetration and victimization (Richards et al., 2017; Wagers et al., 2021), future research on ITFV should also consider bi-directional IPV when it comes to exposure to parental IPV and children’s later IPV experiences. Further, studies often presume males as IPV perpetrators and females as victims in the context of heterosexual relationships; however, that is not always the case. Future studies in this area should use more gender-inclusive approaches.

### 5.2. Considering the Multifaceted Nature of Violence

Multidimensional aspects of violence and abuse, such as duration, severity, context, and frequency, have not been thoroughly considered in the existing literature. For instance, IPV perpetration has often been operationalized as violent actions occurring within the previous 12 months (Ehrensaft et al., 2003; Roberts et al., 2010). Future research should consider a broader scope of IPV experiences rather than focusing on a narrow timeframe in order to capture a person’s entire IPV experience (Kwong et al., 2003). Future studies also should include more characteristics of violence—such as the onset of the incident and chronicity of violence—in considering various facets of CEFV and IPV. This approach can help capture violent experiences in a more nuanced way and promote a more in-depth understanding of these complicated experiences (Kwong et al., 2003).

### 5.3. Considering Socioecological Factors

Numerous studies have examined the links between CEFV and IPV perpetration in adulthood based on various theoretical frameworks (e.g., social learning theory, emotional regulation framework). With some limitations, studies on ITFV found possible mediators and moderators that may explain the pathway from CEFV to IPV perpetration and alleviating factors that may break the cycle of violence. However, most studies have focused on individual characteristics as a mediator or moderator (e.g., attitude towards using violence, emotional regulation; see Table 1). Nonetheless, violence in families does not occur in a vacuum but may be deeply intertwined with various contextual factors (e.g., family, school, work, and community). For instance, research indicates that pathways from CEFV to later violence may be impacted and shaped by an interplay of various factors across individual (e.g., health, disabilities), familial (e.g., parental marital status, family dynamics), and community-related domains (e.g., housing, neighborhood, religious communities; Southern & Sullivan, 2021). Thus, other possible mechanisms of the cycle of family violence should be investigated based on a more holistic and integrative view, drawing on theories of multilayered ecological systems surrounding children and families, rather than solely concentrating on individual traits. Furthermore, while individuals are nested in various ecological domains, the weight of each domain may have a different magnitude of effect on violence. Hence, ITFV literature needs to account for those factors, particularly the ways in which multiple risk factors and protective factors contribute to solidifying or weakening the transmission of violence.

### 5.4. The Need for Advanced and Varied Methodological Approaches

A few recent studies examining ITFV have considered poly-victimization or various patterns of IPV to capture such violent experiences more comprehensively (e.g., Voith et al., 2017; Jung et al., 2019). Enhanced statistical methods, such as a person-centered approach (e.g., latent class analysis), can further expand the knowledge of these complex aspects of ITFV. Future studies can utilize these methods to further investigate the cycle of violence and provide a more thorough understanding of its multifaceted nature.

Despite the utility of qualitative research methods for studying complex interpersonal phenomena, there has been limited qualitative exploration of the ITFV. Existing qualitative research provides valuable insights into the context of a violent incident, the complexities of abuse, and interpretations of their own IPV experiences (Antle et al., 2020; Vilches et al., 2025). To deepen the understanding of ITFV, future studies should use qualitative methods to continue to explore people’s attitudes toward past abusive incidents and how individuals perceive the impacts of these experiences on their violent behaviors.

## 6. Conclusions

The ITFV provides an explanatory model for the pathways through which family violence is perpetuated across generations, offering insights for both research and intervention. Multiple theoretical perspectives provide complementary insights into the pathways linking CEFV and later IPV perpetration. Social learning theory highlights the role of behavioral modeling, suggesting that children who experience family violence are likely to replicate these behaviors in adulthood. Social information processing theory adds that the ways children interpret and respond to social cues mediate the ITFV. The emotional regulation perspective emphasizes the pathways through which early exposure to violence can disrupt emotional development, increasing the risk for aggressive and dysregulated responses in intimate relationships. The stress sensitization theory aligns closely with the emotional regulation framework, as early stress exposure may disrupt the development of adaptive emotional regulation capacities, leading to heightened emotional reactivity and maladaptive responses to later stressors. Evidence from developmental psychopathology research further indicates that adolescent behavioral problems serve as a critical mediator in the pathway from CEFV to adult IPV. Finally, the ACE framework explains that cumulative traumatic experiences lead to later IPV perpetration. Collectively, these findings underscore that ITFV is a multifaceted process, influenced by behavioral, cognitive, and emotional factors. They highlight the necessity of interventions that address multiple mechanisms, including modeling healthy relationships, promoting emotional regulation skills, and mitigating the long-term effects of childhood trauma, to prevent the continuation of violence into adulthood.

Since the late 1980s, research has focused on whether child abuse or parental IPV exerts a stronger influence, and whether such effects are transmitted through same-gender modeling (i.e., fathers influencing sons and mothers influencing daughters) or generalized modeling that affects children regardless of gender (Avakame, 1998; Eriksson & Mazerolle, 2015; Kalmuss, 1984; Kwong et al., 2003). More recently, however, studies have adopted more advanced and comprehensive methodologies to examine how different CEFV types are linked with various IPV outcomes by gender as well as through which gender-specific mediating mechanisms these effects operate (Chen et al., 2025; Jung et al., 2019). Overall, these studies consistently provide at least partial support for the ITFV.

While existing research has significantly advanced understanding of the pathways from CEFV to adult IPV perpetration, gaps persist in addressing the multidimensional and contextual nature of violence. Most studies continue to rely on the victim–perpetrator binary and focus on individual or family-level characteristics, limiting the comprehension of broader systemic and community influences. Moving forward, research needs to adopt a more holistic approach that incorporates socioecological perspectives, recognizes the overlapping experiences of victimization and perpetration, and accounts for the multidimensionality of violence, including severity, frequency, and context of violence. By embracing diverse methodological strategies and theoretical frameworks, future studies can deepen understanding, inform more effective prevention strategies, and ultimately contribute to breaking the cycle of family violence. This review focused on empirical studies examining mechanisms and patterns of ITFV, but future reviews should synthesize studies on protective factors or resilience processes that may mitigate the impact of CEFV on the likelihood of IPV.

## Figures and Tables

**Table 1:** Studies on the Mediating and Moderating Mechanisms of ITFV.

Theoretical Frameworks	Mediating and Moderating Mechanisms
Social learning theory	Attitudes justifying IPV (Eriksson & Mazerolle, 2015; Markowitz, 2001; Wareham et al., 2009)
Social information processing	Two social information processing stages: response generation and response evaluation (Fite et al., 2008)
Emotional regulation perspective	Emotion dysregulation or emotional regulation difficulties (Kim et al., 2009; Oliveros & Coleman, 2021)
Stress sensitization theory	Past-year stressors (Roberts et al., 2011)
Developmental psychopathology	Externalizing behavior problems (Ehrensaft et al., 2003; Low et al., 2019)

**Table 2:** Studies on the patterns of ITFV.

Patterns	Studies	Samples	Methods	Study Results
Gender-specific	Jung et al. (2019)	Two county child welfare agencies of cases (*n* = 457)	A joint model where the latent class model and the latent class regression model were combined	Males who were physically and emotionally abused as children were at increased risk of being involved in multiple types of IPV with intimidation. In contrast, females who were sexually abused were more likely to report multiple types of IPV perpetration and victimization with intimidation.
	Chen et al. (2025)	A sample of males and females in Japan (*n* = 483)	Structural equation modeling	For males, childhood physical abuse directly predicted adult IPV perpetration. In contrast, for females, childhood physical abuse was associated with IPV psychological victimization indirectly through psychological distress.
Role-specific	Kalmuss (1984)	A nationally representative sample (*n* = 2143)	Log-linear analysis	Childhood exposure to male-to-female IPV has been linked to adulthood IPV victimization and perpetration among both genders. The logit coefficient for exposure to parental IPV was significantly larger than the coefficient for child abuse.
Both gender-specific and role-specific	Eriksson and Mazerolle (2015)	A sample of male arrestees (*n* = 303)	Logistic regression	Childhood exposure to male-to-female IPV and bidirectional IPV was linked with later IPV perpetration. More specifically, bidirectional IPV among parents was linked with a fivefold increase, and male-to-female IPV was linked with a threefold increase in the likelihood of IPV perpetration. Exposure to female-to-male IPV and child abuse victimization was related to IPV perpetration.
Generalized modeling	Avakame (1998)	A nationwide probability sample (*n* = 2143)	Structural equation modeling	Fathers’ corporal punishment directly increased the likelihood of physical IPV for males as well as psychological IPV for both genders. Exposure to parental male-to-female IPV directly increased females’ physical IPV.
	Kwong et al. (2003)	A community sample (*n* = 1249)	Hierarchical regression	All types of family violence exposure during childhood were linked with all types of adulthood IPV; childhood exposure to male-to-female IPV has been linked to adulthood IPV victimization as well as perpetration among both males and females.

## Data Availability

No new data were created or analyzed in this study.
